# Data-driven measures to mitigate the impact of COVID-19 in South America: how do regional programmes compare to best practice?

**DOI:** 10.1093/idpl/ipab002

**Published:** 2021-03-01

**Authors:** Taís Fernanda Blauth, Oskar Josef Gstrein

**Affiliations:** 1 Taís Fernanda Blauth, University of Groningen, Campus Fryslân, Leeuwarden, The Netherlands, E-mail: t.f.blauth@rug.nl; 2 Oskar Josef Gstrein, University of Groningen, Campus Fryslân, Leeuwarden, The Netherlands, E-mail: o.j.gstrein@rug.nl

## Abstract

• This article analyses data-driven measures used in South America to mitigate the impact of COVID-19. Based on a broad review of relevant programmes in the region three selected cases from Argentina (Cuidar App), Brazil (use of personal data by *IBGE*), and Chile (CoronApp) are evaluated against best regional and international practices.

• Our findings suggest that programmes in South America mirror approaches in other global regions and as such face many similar challenges. There is no clearly defined purpose, a lack of transparency, and the need for readjustment soon after initial development.

• While the region is heavily affected by COVID-19, the three case-studies analysed demonstrate that policy makers in the region failed to establish trust in the measures. This can be deducted from low penetration rates of the programmes in Argentina and Chile.

• Finally, there are serious concerns regarding the long-term impact of these programmes upon human rights (especially privacy) and human dignity.

## Introduction

The SARS-CoV-2 virus started to spread quickly from a food market in the city of Wuhan, China, around December of 2019.^1^ Following the declaration of a pandemic by the World Health Organization (WHO) on 11 March 2020,^2^ COVID-19 has reached every corner of the world. By 29 July 2020, more than 16.7 million cases have been confirmed, and the number of deaths surpasses 660,000 worldwide.^3^ South America is among the regions that have been affected particularly, with Brazil, Chile, and Peru among the top ten nations for confirmed cases.^4^ While states have taken several measures to contain the virus—including lockdowns and travel restrictions—many governments are exploring innovative strategies to deal with the pandemic. As vaccines and effective treatment are still under development,^5^ governments, private organizations, and scientific institutions develop data-driven programmes as an option to mitigate the impact of the pandemic.^6^

These are either designed by governments themselves, based on initiatives by corporations such as Google and Apple^7^, or have been developed by researchers and activists.^8^ For example, in a case in China facial recognition cameras identified a man who refused to stay in quarantine, leading authorities to alert his employee as a warning.^9^ In Spain, drones were used to replicate audio messages encouraging the population to stay at home during partial lockdown.^10^ In the United States, companies are looking at social distancing detectors to be implemented in the workplace.^11^ Researchers in many countries have also been delving into the development of models that apply AI techniques to predict diagnosis and/or prognosis of developments around COVID-19.^12^ However, these measures result in concerns about an unprecedented degree of public and private surveillance potentially undermining individual and collective autonomy.

Although apps and other data-driven solutions^13^ can potentially play a relevant role in managing the pandemic, three different but interconnected issues must be taken into consideration. First, most of these efforts are experimental and as such might not be effective for the intended purpose, if the purpose is clear at all. It is important to note that not only the technical design of systems is relevant, but also the strategy for integration and mass-adoption of society, taking into account potential spill-over and side effects. This can be exemplified by the development and deployment of a digital contact tracing app in Iceland. Although nearly 40 per cent% of the residents of the country had downloaded the app, manual contact tracing is still necessary, and it remains questionable how much the use of a digital surveillance system has added in terms of efficiency. ^17^ Secondly, when discussing solutions that rely on digital infrastructure, one has to consider the significant part of the population which does not have Internet access, or possess state-of-the-art smartphones and computers. As discussions in Germany have shown,^18^ this situation could enhance the digital divide between the wealthy and deprived, provoking an ample gamut of problems. Thirdly, there are serious concerns from the perspective of data protection and privacy, since the collection, storage, analysis, and sharing of data can be subject to abuse. This is a topic of special concern that has consequently resulted in several initiatives being launched, such as the COVID-19 Digital Rights Tracker^19^ and Pandemic Big Brother.^20^ These map digital rights restrictions and violations caused by the implementation of different technology initiatives during the pandemic. Effective processes and design elements guaranteeing privacy by design and by default are necessary to build trust. If applied correctly, such technical and organizational elements can enhance acceptance of data-driven measures among the population, potentially increasing effectiveness as well.^21^

In this article, we consider these aspects by analysing and discussing three data-driven programmes in South America to mitigate the pandemic. The article is structured as follows. In the ‘Methodology’ section, we describe the methodology of the study, including the research questions and criteria for case selection. In the ‘Guiding principles and best practices’ section, we describe and analyse best practices identified by experts, as well as lessons learned in similar systems in other countries around the world. We then describe the three selected cases, followed by a comparative table of the measures in the ‘Discussion’ section, where we also discuss the effectiveness of the three chosen programmes. Finally, we conclude discussing whether human rights, including privacy, are respected, protected, and promoted through them. Based on our findings we suggest that even in times of emergency and uncertainty, adherence to fundamental rights, sound guiding principles, and rule of law are essential to develop a coordinated response.

## Methodology

This article aims to investigate how data-driven programmes to mitigate the pandemic in South America compare to best practices. We focus on (i) the effectiveness of the measures, and (ii) how the programmes respect, protect, and promote human rights with a particular emphasis on privacy. In addition, we seek to answer the following sub-questions:

- What are the guiding principles that should be considered when developing and/or implementing data-driven initiatives to manage the COVID-19 pandemic?
- What are the main initiatives proposed and/or implemented by South America’s governments?
- Do South American governments comply with guiding principles in the development and/or implementation of data-driven initiatives to manage the pandemic?

To identify the most useful case-studies presented in this article, we compiled a list of smartphone applications (apps) and other data-driven initiatives aimed at managing the population in response to the pandemic. These were either developed by a South American government or adopted by it. In total, 14 initiatives in nine sovereign states were identified. Information regarding the specific aims and basic functionalities was collected, as well as other specific data on the country (eg Gross Domestic Product in 2019), and the number of positive cases of COVID-19. In detail, the criteria for selecting three cases for analysis and discussion were based on the following indicators: (i) GovTech Index, which measures the maturity of GovTech ecosystems in Latin America;^22^ (ii) Economy—GDP per millions of US$;^23^ (iii) Population;^24^ and (iv) Number of Positive Cases of COVID-19 on 21 June 2020.^25^ The countries which have higher results in these indicators, combined, are Brazil, Colombia, Chile, and Argentina, respectively. As a next step, a case-by-case analysis was conducted to identify which of the measures from these countries were best-documented with sufficient amounts of accessible and reliable sources, enabling thorough analysis and discussion of each individual case. In this phase, the Colombian programme was excluded from the study due to insufficient information.

As a result, we focus on the data-driven programmes in Chile (CoronApp) and Argentina (Cuidar App). Furthermore, four relevant cases were found in Brazil: the use of personal data by the *Instituto Brasileiro de Geografia e Estatística—IBGE* (Brazilian Institute of Geography and Statistics), the *Coronavírus SUS* App, the *Auxílio Emergencial* App, and the *Caixa Tem* App. From these, the first case was chosen due to its particular relevance in the current scenario. The measure provoked questions regarding its constitutionality, and when the Supreme Court scrutinized the legal framework the programme was indeed declared unconstitutional.

Finally, we identified several concrete and detailed guiding principles and/or recommendations for governments developing data-driven measures, published by regional (ie Latin America)^26^ and international^27^ organizations. These documents served as a foundation for determining best practices.

## Guiding principles and best practices

Apps and other data-driven measures aimed at mitigating the impact of the pandemic should not be deployed in a vacuum of guidance. General principles can be helpful to avoid and identify abuse by authorities. The UN High Commissioner for Human Rights^28^ and civil society^29^ urged states to maintain a human rights-based approach throughout. Hence, responses have to be non-arbitrary (eg publicly declaring the eventual use of emergency powers), necessary, proportionate, as well as non-discriminatory.^30^ They should not be used as a way to illegitimately target specific groups and individuals or violate their fundamental rights.

As states navigate uncharted territory, it might be particularly useful to consider lessons learned from using innovative data practices in the Humanitarian sector, as well as to look at the best practices establishing ‘data responsibility’.^31^ Measures for proximity and contact tracing, symptom checking, quarantine compliance, and flow modelling resonate in numerous social, ethical, and legal issues. These evoke challenges relating to individual and collective autonomy, justice,^32^ solidarity, beneficence, and non-maleficence, which need to be addressed throughout all stages of design, deployment, and evaluation of measures.^33^

Drawing on conditions established by civil society organizations to prevent illegitimate COVID-19-related digital surveillance,^34^ as well as consideration of recommendations for tracking and tracing apps established by the Inter-American Commission on Human Rights,^35^ the World Health Organization,^36^ the European Data Protection Board,^37^ the American Civil Liberties Union,^38^ and the Electronic Frontier Foundation,^39^ we compiled a list of best practices that can guide the design of data-driven measures to mitigate the impact of COVID-19:

- Voluntary use: a fundamental requirement for applications is that individuals should be able to decide whether they want to download and use apps and other systems through opt-in. In addition, explicit and individual consent is required, or a dedicated legal framework justifying and guiding the execution of a data-driven measure. It is particularly important to establish in such a framework how concerns relating to privacy and data protection are addressed.^40^ People should not experience negative consequences for not using the app, such as not being able to go to work or be denied services from a government or private parties. This also means that an individual should be free to delete the application and the data that may have been stored without any consequences.
- Purpose limitation: the data shared through the apps/systems should be collected and used solely for legitimate public health goals that must be clearly and specifically described. Sharing or using such data for other purposes, be them commercial, political, or (national) security should be avoided to prevent abuse and improve the likelihood of general acceptance.
- Data minimization: apps and other systems should collect, process, and store as little data as absolutely necessary to fulfil the public health aim. This also means that the purpose has to be clear from the outset.
- Exit strategy: whenever the emergency has ended, developed systems should be terminated and personal data should be deleted. Specific measures developed to manage the pandemic should be time and purpose-bound. Hence, safeguards should be in place against mission creep. It is essential to clearly indicate who will determine the continuity, termination, or improvement of the existing emergency framework, by when and under what criteria.^41^
- Transparency: development of data-driven measures should be carried out with accountability kept in mind. This means that governments should be transparent about the policies in place and about what type of data is being collected by whom, for which means, and how it is being used. Transparency is necessary for people to understand how the programmes operate, which data is stored, and why. This enables individuals to make an informed decision on whether they want to use the app or participate in a program. The fear or suspicion of surveillance might prevent individuals from participating, which could undermine effectiveness. Additionally, the source code of applications should be publicly available for third-party audits and independent experts to review, and data protection impact assessments should be carried out when developing programmes. Two noteworthy examples in this regard are the open-source apps of Switzerland and Germany.^42^
- Privacy and data protection by design and by default: data-driven initiatives must respect the principle of privacy and data protection by design and by default.^43^ Some projects, such as contact tracing apps developed in Norway, Bahrain, and Kuwait, have shown an overly invasive approach.^44^ Thus, any initiative must support data anonymization, state-of-the-art cryptographic techniques, and further measures to secure data and prevent harm in case of leaks or breaches.

## Case studies

On 26 February 2020, a case of COVID-19 was confirmed in São Paulo, Brazil. This was the first positive case in Latin America.^45^ Governments of the region implemented measures and established guidelines to manage the pandemic, taking an array of initiatives to protect public health and contain the spread of the virus. Among these initiatives, some countries have developed and/or adopted data-driven solutions as an additional measure to manage the crisis. If not respecting, protecting, and promoting human rights, the impact of such measures might be severe and have long-lasting consequences. This section will describe three case studies: the Cuidar App in Argentina, the use of personal data by the *IBGE* in Brazil, and the CoronApp in Chile.

### Cuidar App in Argentina

In Argentina, starting on 20 March 2020, preventive and mandatory physical distancing was mandated through decree 297/2020, to slow the spread of Coronavirus and protect public health.^46^ On 23 March 2020, the *Secretaría de Innovación Pública* presented a web form and an app for the self-evaluation of COVID-19 symptoms. The programme was criticized for the amount of personal data and permissions it required, which was allegedly not compatible with the aims of the app.^47^ By the end of April, an update was released, which fixed the identified problems.^48^ The current version has two primary purposes.

Firstly, it should be a tool for self-diagnosis of symptoms. Citizens are advised to use this functionality irrespective of whether they have symptoms and, if they show symptoms compatible with COVID-19, the application presents recommendations following the provided individual information. The answers to the self-evaluation form in the app are legally binding declarations,^49^ which means that false declarations or untrue answers to the symptom check might be punished. A message in the app recommends the user to take the self-evaluation test every 48 hours to keep the data accurate and updated. Additionally, the app proposes ways of contacting health authorities. Users who have symptoms might also be contacted by the *Comités Operativos de Emergencia Provinciales—COEPs* (regional health emergency committees), which will provide assistance and necessary information. However, it is unclear whether there is a set of criteria for contacting individuals.

Secondly, the app has the purpose of serving as an alternative for individuals to store the *Certificado Único Habilitante de Circulación—CUHC* and present it to authorities when necessary. This certificate confirms that an individual is allowed to travel and use public transportation,^50^ in accordance with a set of exceptional circumstances.^51^ By default, it is presented as a QR code in the app.^52^ According to the government’s official website, the code will appear if the self-evaluation result is negative for COVID-19 symptoms, allowing the person to be in public space, in accordance with the exceptions mandated by the Argentinian government. People who do not have any symptoms, but do not possess a permission certificate, can only leave their house to shop in nearby markets and shops, following the recommendations for COVID-19 prevention. Those who have symptoms should stay at home and follow the recommendations provided by the system. Even though downloading the app is voluntary, the certificate of circulation is mandatory to roam around freely. Furthermore, if a person cannot use the app, a certificate can be requested on the official government website, which can then be saved as a file on a compatible device, or printed.

The Android version of the app requires numerous permissions: camera access, the ability to send and receive data via the Internet,^53^ full network access with the ability to view network connections, prevention of the device from entering sleep mode, as well as location (GPS and network-based). According to the *Agencia de Acceso a la Información Pública,^54^* the current legislation does not forbid monitoring an individual’s location. Nevertheless, the data processing should be conducted respecting the human right to privacy.^55^ Hence, one may assume that any intrusion must be necessary and proportionate, based on an individual assessment.

The *Subsecretaría de Gobierno Abierto y País Digital* created a database called ‘COVID-19 Ministerio de Salud’ to centralize the data collected through the app.^56^ Such a ‘centralized approach’ means that user information is sent from within the app to the servers of a private company chosen by Argentina’s government.^57^ The data is managed by governmental authorities who can analyse it.^58^ The centralized architecture raised a debate concerning data protection and storage of sensitive information,^59^ which might be better protected by adopting a decentralized architecture where the user’s device is the main point of data processing and analysis.^60^ According to Argentinian personal data protection law, health data falls into the category of ‘sensitive data’, thus enjoying special protection under the current legislation.^61^ For instance, the legislation establishes that adequate dissociation mechanisms (eg encryption, pseudonymization, anonymization) should be in place and that the identity of the individual should be preserved in relation to health data. However, the individual’s identification is mandatory when using the *Cuidar* app^62^ and it is unlikely that anonymization can be performed.^63^

When the first app was launched (app *COVID-19—Ministerio de Salud*), the *Dirección Nacional de Migraciones (DNM)*, Argentina’s immigration agency, announced that travellers to the country would be required to download the app, and keep it activated on their device for at least 14 days.^64^ Thus, a government-supported app was mandatory for every person arriving in Argentina to monitor the compliance to quarantine rules, and the app *Cuidar* is voluntary for those that are already in Argentina.

On 25 May 2020, the government opened a repository page on the platform GitHub,^65^ where the source code of the app would be published. This measure was taken to provide and guarantee transparency since the source code could be audited and revised. However, even though this channel was open and a ‘readme’ file posted, the code was not published as of the time of the writing.^66^ The app has 5 million registered users as of 2 July 2020.^67^

### Use of personal data in Brazil by the IBGE

In Brazil, the law 13.979/2020, published on 6 February 2020, regulates the measures which can be taken in the country regarding the pandemic.^68^ Later, on 17 April 2020, Brazil’s President Jair Bolsonaro published Executive Order 954/2020, determining that telecom operators had to share their customers’ data (names, telephone numbers, and addresses), both of individuals and companies, with the *Instituto Brasileiro de Geografia e Estatística* (called *IBGE* in its Portuguese acronym).^69^ According to the Executive Order (EO), the collected data would be used to conduct interviews to develop national statistics during the pandemic,^70^ and the data would: (i) be confidential, prohibiting *IBGE* from sharing it with private companies or public organizations; (ii) be used for the single purpose of conducting interviews to develop national statistics; and c) not be used as evidence in administrative procedures (legal or tax-related). In addition, it is stated that data shared in the terms of the agreement would be deleted once the public health emergency was over.

In an official note,^71^ the *IBGE* highlighted that the data would be relevant to continue the research efforts being conducted by the organization and for planning responses to the crisis in the health sector. The note stressed the data would be essential to manage the health and economic challenges posed by the pandemic. In an additional note,^72^ on 20 April 2020, the institute re-stated the importance and necessity of the data to enable the development of statistics by conducting non-face-to-face surveys, especially since the demographic census, which was supposed to be conducted in 2020, was postponed due to the pandemic. The note also mentioned that collected data cannot be used for monitoring and tracking users, specifying its use for the sole purpose of conducting research by telephone.

Civil society criticized the EO,^73^ which was later contested in the *Supremo Tribunal Federal* (Brazilian Supreme Court) through five *Ações Diretas de Inconstitucionalidade* (unconstitutionality claims). The procedures were initiated by the *Ordem dos Advogados do Brasil* (Federal Bar Association)—ADI 6387, and four political parties (*Partido da Social Democracia Brasileira—*ADI 6388, *Partido Socialista Brasileiro—*ADI 6389, *Partido Socialismo e Liberdade—*ADI 6390, and *Partido Comunista do Brasil—*ADI 6393).^74^ The arguments involved the unconstitutionality of both the procedure and the substance of the EO. Specifically, three central claims were made on the substance of the EO regarding data protection.^75^ Firstly, that it did not demonstrate a clear correlation between the data that would be shared and its alleged use, violating the principle of purpose limitation. Secondly, that the EO did not provide the reasons behind the need for such data and the way it was going to be used, violating the principle of transparency. Lastly, that the amount of data that was required was not clearly justified,^76^ in addition to the lack of security measures preventing misuse and data breaches, violating the principle of proportionality.

On 7 May 2020, by a majority of 10 out of 11 Justices, the Supreme Court declared the Executive Order 954/2020 unconstitutional, causing its annulment. This decision is considered a milestone for data protection in Brazil,^77^ since the Supreme Court confirmed that data protection has a constitutional fundamental right status, confirming that the principles of purpose limitation, transparency, and proportionality have to be respected in upcoming legislative and administrative acts.

### CoronApp in Chile

The government of Chile has also developed an app as a data-driven initiative to manage the pandemic. It is called ‘CoronApp’ and has four main purposes.^78^ Like the Argentinian app, it offers self-evaluation of symptoms, enables individuals to check their health status and to have access to risk classification. Self-evaluation is done through a series of questions which the user has to answer by either checking boxes (eg if the person has a dry cough), or by filling in information (eg body temperature). Depending on the gravity of symptoms, the app might suggest visiting a health care centre. Each user can take the test every hour.^79^ It is noteworthy that health data is considered sensitive data according to Law 19.628. According to Article 10, sensitive data cannot be processed unless (i) the law authorizes it, (ii) the owner consents to it, or (iii) it is necessary for granting a health benefit to the owner.^80^ However, the current legislation does not indicate public health emergencies as grounds for processing such data. In fact, the pandemic highlighted numerous gaps in the existing law, especially regarding the management of sensitive personal data.^81^

The second purpose of the app is to serve as an information channel between the government and population. It acts as medium to receive notifications from the Ministry of Health, to deliver informative content regarding the measures and evaluation of the pandemic, and to provide contact information of health centres. Thirdly, the app allows the adding of information regarding the user’s quarantine, such as start and end date, as well as the address of quarantined individuals. In this case, the app will also send a notification if a person leaves the permitted area.^82^ Fourthly, the app is a tool to inform and/or denounce high-risk behaviours or events. Users can notify the government if they identify agglomerations, noncompliance of quarantine rules, or if they see long queues for services.

To start a session in the app, a user—who has to be over 18 years old—has two options.^83^ One can either enter the *Clave Única*, which is a unique password used to access digital services offered by the government, serving as digital identity.^84^ Otherwise one has to create a profile using personal documents. For instance, it is possible to register using the unique tax registry (*RUT* number) and the number of the ID, in which case it will also be necessary to fill in information such as the telephone number, age, and region of residence. In the additional menu users can edit personal information, access a glossary, check frequently asked questions, contact the digital branch of the government, and others.^85^ There is also an option to add dependent users, up to a maximum of eight people.^86^ People who still have questions or need additional advice can use a virtual assistant based on ‘Artificial Intelligence’, which is available for general consultations through WhatsApp, or call the centre for health assistance.^87^ The privacy policy of the app was criticized for the use of imprecise terms, as well as for containing relevant gaps such as not explicitly clarifying the basis for the legitimacy of processing the data collected by the app.^88^

The chief of the *División de Gobierno Digital de SEPREGS*, Francisco Rodríguez, highlighted that the CoronApp should be seen as an opportunity to include the elderly in the digital world.^89^ Since people would stay at home, he argued, tech-savvy citizens could teach the older generation how to use the app. The app is available on the App Store (iOS) and Google Play (Android), and by the end of May it was reported that less than 1 per cent of the Chilean population had downloaded the app.^90^

## Discussion

In Table 2,^91^ we summarize the main characteristics of the three applications with a particular focus on how they perform in the criteria outlined in the best practices section.

**Table 1.** Data-driven tools against COVID-19

Tool/Applications	Purpose	Instances documented for^14^
Quarantine Enforcement through Data Collection	To be informed about compliance with quarantine measures.	China, Hong Kong, Taiwan, Singapore.
Digital Contact Tracing	To identify those who might have been exposed to COVID-19, so that they quarantine themselves and stop further dissemination.	Ecuador, Brazil, Singapore, South Korea, India, Germany, France, United States, Canada, and many more.^15^
Flow Modelling	To identify how many people pass through certain locations and how quickly.	United States.
Social-graph Making	To identify which individuals tend to meet repeatedly.	No country currently.
Digital Immunity certificates	Certificates (or ‘passports’) for individuals who are immune against COVID-19.	No country currently.
Communication and Information	To provide information to the population about COVID-19.	Peru, Bolivia, Chile, Brazil.
Self-evaluation	To assess if individuals have COVID-19 symptoms.	Argentina, Chile, Peru.
Contact Platform	To provide an additional platform for contact between individuals and health authorities.	Argentina, Chile, Uruguay.
Financial Support	For individuals to request financial support.	Brazil.

Adapted from *The Economist*.^16^

**Table 2.** Case studies evaluated according to guiding principles and best practices

	Argentina’s Cuidar App	Brazil’s Use of Personal Data by *IBGE*	Chile’s CoronApp
I. Purpose	Self-evaluation tests and ‘wallet’ for the circulation certificate.	Statistics	Informative, self-evaluation, quarantine information, and denounce situations of risk.
II. Voluntary use			
(II.a) Opt-in	Yes, except for travellers arriving in the country.	Not applicable	Yes
(II.b) Sanctions for non-participation	No, except for travellers arriving in the country.	Not applicable	No
III. Purpose limited to COVID-19 management	Yes	No	Yes
IV. Data minimization	No^93^	No^94^	No^95^
V. Exit strategy	Personal data will be eliminated; ‘aggregated anonymised’ data will be kept for statistical purposes.	Data will be deleted from IBGE’s database when the ‘emergency situation’ ends.	Personal data will be eliminated; ‘aggregated anonymised’ data will be kept for statistical purposes.
VI. Transparency			
(VI.a) Policy transparency	Yes	No (eg storage of data and security of database are unclear)	Yes
(VI.b) Open-source code	Not available at the time of writing.	Not applicable	No
VII. Privacy and data protection by Design			
(VII.a) Measures in place to guarantee security of data	Data is encrypted and only authorized users have access.	Unclear	In accordance with the Ministry of Health’s information security policies.
(VII.b) Storage	Cloud—third party (Amazon Web Services)	Unclear	Cloud—third party (Amazon Web Services)

### Effectiveness to mitigate the pandemic

Without a clearly defined purpose it is difficult to assess the effectiveness of such programmes; it is not always clear what they address, or try to achieve. On the one hand, information gathered through surveys*—*such as in the Argentinian or Chilean case*—*might not be necessary or useful in order to find out whether one really has symptoms relating to COVID-19. On the other hand, implementing such new systems creates comprehensive data trails, which need to be carefully managed to protect the privacy and the autonomy of individuals and groups. Certainly, understanding the characteristics of the virus and the corresponding disease is an ongoing process and it is therefore difficult for governments to identify the right and most effective measures.

However, it is also important not to overstate the usefulness of such initiatives. Considering to what extent data-driven measures are effective is necessary to evaluate whether they should be developed in the first place. Otherwise, investment in these might be considered inadequate since more practical measures could have been taken, such as investing in manual contact tracing or medical equipment. Additionally, developing technological solutions to mitigate the impacts of the pandemic should not be done to serve political interest alone. Such programmes must not be a way for governments to merely attest that ‘something is being done’, or to use the initiative as a way of avoiding blame in case of a backlash.^92^ A justifiable, well-designed, and principle-based app should be part of a wider response strategy.

Notably, the success of applications developed to mitigate the impact of COVID-19 largely depends on the cooperation of the population. For contact tracing apps, it is estimated that approximately 60 per cent of a country’s population (or around 80 per cent of the smartphone users) have to use them to be clearly effective. ^96^ However, as of December 2020 even the *TraceTogether* app used in Singapore (and mandatory for all residents) has only been downloaded by 57 per cent of the population,^97^ which is still short of the 60 per cent penetration rate considered important for tracing apps to be fully effective. Similarly, a contact tracing app in Iceland, which was recognized for having one of the highest penetration rates of tracing apps worldwide, was adopted by approximately 40 per cent of the population.^98^ Since high adoption rates are crucial for the apps to work effectively, and the principle of voluntary use should be preserved in democratic countries such as Argentina and Chile, it is necessary to consider which factors might impact and increase acceptance and consistent use.

One of those is the level of trust and confidence individuals have.^99^ The low number of downloads of the app in Chile (less than 1 per cent of the population^100^), for instance, might be evidence of the lack of trust of the population in the programme. Civil society organizations have reported on privacy issues, data protection risks, and the ineffectiveness of the app, which could have influenced public opinion regarding the programme.^101^ Additionally, the app does not follow best practices in terms of transparency and data minimization. This means that fear of surveillance, a lack of clear policies, or doubts about usefulness, as seen in the Chilean case, might undermine user confidence. Finally, a point of concern might also be that the system relies on third-party cloud services which are offered by Amazon*—*a powerful corporation outside national territory. Hence, principle-based solutions that take all of these factors into account as well as respect, protect, and promote human rights could pave the way to higher adoption rates.

Furthermore, the socioeconomic reality of South America and the population’s digital access are important. One has to consider whether such programmes and advanced technologies correspond to the places where they will be used, also considering potential cultural differences. The issues in this regard are threefold. Firstly, a large part of the population does not have access to reliable digital infrastructure (or no access whatsoever). The pandemic has made problems related to the digital divide even more apparent since many people were prevented from accessing information or continuing their activities, such as studying, due to the absence of connectivity.^102^ Moreover, digital inequalities can enhance the risks of vulnerable groups being exposed to the virus.^103^ Secondly, many do not have adequate devices to download and use the apps since some applications might only run on the latest versions of the operating systems.^104^ This means that even individuals who have connectivity might not be able to use the apps, simply because they do not have a smartphone running the latest Android or iOS systems. Thirdly, the lack of basic digital skills is still evident in most countries, which might prevent people from adequately using the app. In the European Union, for instance, 85 per cent of the population used the Internet in 2019, but only 58 per cent have at least basic digital skills.^105^ In Latin America digital literacy is also a known challenge.^106^ It is noteworthy that the government of Chile encouraged younger citizens to help the older generation to use the app, which can be seen as a strategy to increase both its usage and improve people’s digital skills. As a result, the lack of access^107^ and digital skills might cause lower adoption rates of apps designed to mitigate the effects of the pandemic, furthering the digital divide.

It is also crucial to analyse whether the functionalities of the measures are being used and performing well. The COVID Symptom Study app, available in the United States and United Kingdom, for instance, has shown interesting results for the preventive self-evaluation of symptoms.^108^ However, as the Argentinian government recommends, the test should be taken frequently to keep the data updated and accurate. Nevertheless, only 5.5 million self-evaluation tests were performed, while the app has had 5 million downloads.^109^ This can be a sign that the users are not following the proposed guidance.

The low number of re-taken tests in Argentina illustrates another problem, which is the false sense of security. The population might feel safer assuming that people are regularly checking their symptoms and thus staying at home in case of suspicion. However, this is not the case. Even those who have downloaded the app (around 11 per cent of the population^110^) are not necessarily using its main feature frequently, which risks making the app ineffective.

In addition, the Argentinian policy of allowing certain individuals to leave their house as long as they have a certificate of permission can also be questioned. If a person is authorized to have a certificate, it can be displayed on the smartphone if the self-evaluation test is negative for symptoms of COVID-19; if positive, the certificate cannot be shown as a QR code.^111^ This strategy has two main drawbacks, the first of which is related to effectiveness. Although evidence suggests that infected individuals not displaying symptoms might be less likely to transmit the virus, it is still possible that they can transmit the disease according to the current understanding of the transmissibility.^112^ Hence, if a person is infected and asymptomatic, this will result in a symptom check which will allow them to leave their house (with a certificate), thus potentially transmitting the virus to others. The second drawback refers to the possibility of people cheating on their self-evaluation tests to be allowed to go outside. Even though the form used for the evaluation has a legal character, there is no guarantee that all people will sincerely cooperate, and it is unclear how non-compliance is enforced in practice.

Similar approaches were identified in other countries. In some places in China, for instance, access to certain public places is managed through a QR code system, which classifies individuals as ‘green’ (allowed to circulate), ‘yellow’ (should be quarantined), or ‘red’ (high risk, police could intervene).^113^ It remains largely unclear and opaque how this assessment is made,^114^ differently from the Argentinian app which is based on the self-evaluation test. In addition, the creation of an immunity certificate (or ‘immunity passport’) has been discussed. Such a certificate enables individuals to go to work, study, and travel. It would be awarded to those who have antibodies against SARS-CoV-2, assuming that they would be protected against a new infection.^115^ However, since scientific evidence on how immunity can be established and how long it lasts is still missing, the adoption of this measure is currently not recommended. Furthermore, even if the scientific knowledge on immunity would be sufficient, issues of discrimination and social injustice might arise.^116^

Finally, the analysed South American data-driven programmes have a low adoption rate, a common problem among the solutions designed during the pandemic around the world. The lack of Internet access and/or digital skills might be one of the reasons for missing cooperation of the public. However, trust is also undermined by the programmes’ excessive collection and use of personal data, which is combined with non-transparent policies and behaviour. Those factors should be taken into consideration when developing technological solutions. A poor solution might be worse than an ineffective one as it can create negative long-term effects, resulting in persistent human rights violations undermining human dignity (eg in case of permanent and ubiquitous surveillance), or create harms for public health through evoking a false sense of security.

### Respect, protect, and promote human rights

According to the United Nations and numerous resolutions on privacy in the digital age in particular,^117^ states as well as private corporations ought to respect, protect, and promote human rights in the digital age. Nevertheless, the rather abstract global framework of the United Nations needs to be regionally adapted and interpreted to be applicable and enforceable in specific national cases, such as the one in Brazil.^118^ During the pandemic, Brazil’s President edited an EO that caused debate regarding its constitutionality and the possibility of violation of data protection rights. In addition, it was not clear how the statistical research proposed in the EO could help to deal with the pandemic, causing concern about the necessity of the measure in the first place. Even though the country has a legal framework that can support data protection claims, the main specific legislation on the topic, the Brazilian General Data Protection Law, had not entered into force by the time the EO was edited. With serious concerns regarding administration and enforcement of the data protection framework, some practical aspects were unclear.^119^ The law was supposed to become enforceable in August 2020 but, due to the pandemic, was initially postponed to 2021.^120^ Subsequently however, in September 2020, the legislation entered into force,^121^ regardless of the existence of unclear provisions and lack of an effective DPA in place. Despite specific legislation at the time, the case described in the previous section was taken up by the Supreme Court and the importance of the personal data was highlighted by the justices. The Brazilian case study can be considered a victory for human rights, demonstrating that not everything is justifiable in the midst of a health crisis.

The illegitimate collection of large amounts of data is not a problem restricted to Brazil. The self-evaluation of the symptoms functionality in the Chilean app has been criticized because it unnecessarily aggregates health data (such as pre-existing pathologies and travels to risk areas) with personal data (such as name and phone number), which is sent to the authorities for health care decisions.^122^ The relevance of the identification of individual data is unclear since the app could deliver recommendations regardless of personal data, which would be as effective as the procedures that are in place. In other words, there is no function for individual identification in the tests, which only raises the risk of exposing citizens’ data, subjecting them to possible violations of privacy and discrimination.^123^

The Singaporean *TraceTogether* app also received some criticism for unnecessarily sharing too much information with the government.^124^ Authorities have a centralized database that links the app identifiers to the user’s contact information. When someone tests positive, authorities can check the identifiers the person has come in contact with as well as associated phone numbers and e-mail addresses. This information is then used to notify those who have been exposed. This creates a high level of risk if all governmental entities—or third-parties—gain access to this information. In the Chilean case, keeping the population informed and aware could alternatively be done in a non-personalized form by suggesting preventive measures in general, as well as displaying information about health care centres in the individual’s region (rather than the centres closest to the user’s specific location). Nonetheless, the app collects a large amount of personal data.^125^ Finally, an excess of data shared through an app was also identified in the Argentinian case.^126^ A change in strategy, namely considerably reducing the amount of collected data, would fit more adequately with the best practices identified, namely those of protecting privacy and data protection, thus making the apps a more trustworthy solution.

After analysing the South American programmes, it is possible to conclude that the legal framework and institutions are in principle able to provide essential protections to the rights to privacy and data protection. Brazil’s case shows that, even when there is illegitimate use of personal data, it is still possible to resort to legal action and deem a measure invalid. Nevertheless, the Argentinian and Chilean programmes are not living up to best practices and similar apps in other countries seem more trustworthy (ie open-source code publicly available, such as in Germany’s example^127^).

## Conclusion

The Coronavirus pandemic is a severe test for the resilience of societies across the world. In the search for exit and mitigation strategies, it has sparked the development of innovative data-based programmes. We found that data-driven approaches in South America mirror programmes in other regions of the world to a high degree. Furthermore, they face similar challenges such as low adoption rates due to the difficulty of creating trust, stemming particularly from enduring questions around compliance with human rights requirements and the rule of law. The cases in Argentina, Brazil, and Chile show that measures are often rushed and have to be adapted or reconsidered soon after being deployed. The development and deployment of such data-driven approaches are immensely complex. Intertwined ethical, legal, and social aspects have to be taken into account and addressed through the design of technological and organizational oversight elements. If trust cannot be created during development and deployment, citizens refrain from using applications and systems, which renders the tools ineffective.

Based on the analysed principles and best practices, it is possible to draw general recommendations to enhance trust in technological measures and to avoid overly invasive digital surveillance in South America: (i) use of tools and applications should be voluntary, without sanctions for non-participation; (ii) the purpose of the measure should be limited to COVID-19 management; (iii) the data collected should be minimized as much as possible for the function of the system; (iv) government should establish how and when the measure will be terminated; (v) transparency of the policy and the code should be guaranteed, enabling external audit and clarity of the function of the system; and (vi) privacy by design and data protection by default should be at the centre of any strategy. Even in times of emergency and uncertainty, adherence to fundamental rights, guiding principles, and the rule of law are essential to be able to develop a coordinated response to such an overwhelming crisis.

Finally, the cases in this study also suggest that not all problems can or should be solved by ‘coding an app’, especially since not everyone has access to the necessary infrastructure to be part of this type of solution. While technology might be helpful to mitigate the impact of a pandemic, it will only be effective as part of a wider societal strategy, which has to be transparent and serve clear purposes. This might be the most useful insight to create better responses to similar crises in the future.

## Footnotes

1 ‘Coronavirus Disease 2019 (COVID-19)’ (World Health Organization, Geneva 2020) Situation Report – 94 <https://www.who.int/docs/default-source/coronaviruse/situation-reports/20200423-sitrep-94-covid-19.pdf?sfvrsn=b8304bf0_4> accessed 6 July 2020.

2 ‘Coronavirus Disease (COVID-19): Events as They Happen’ (World Health Organization, Geneva 2019) <https://www.who.int/emergencies/diseases/novel-coronavirus-2019/events-as-they-happen> accessed 7 July 2020.

3 ‘COVID-19 Map’ (Johns Hopkins Coronavirus Resource Center, 2020) <https://coronavirus.jhu.edu/map.html> accessed 29 July 2020.

4 ibid.

5 ‘Draft Landscape of COVID-19 Candidate Vaccines’ (World Health Organization, Geneva 2020) <https://www.who.int/publications/m/item/draft-landscape-of-covid-19-candidate-vaccines> accessed 6 July 2020.

6 ‘Data Protection Law & Covid-19: An Observatory’ (Law, Science, Technology & Society Research Group, 13 July 2020) </en/data-driven-approaches-to-covid-19-data-protection-law-dpl-x-covid-19> accessed 13 July 2020.

7 ‘Exposure Notifications: Using Technology to Help Public Health Authorities Fight COVID-19’ (*Google*) <https://www.google.com/covid19/exposurenotifications/> accessed 24 June 2020.

8 ‘DP-3T—Documents’ (*GitHub*) <https://github.com/DP-3T/documents> accessed 8 July 2020; Michael Veale, ‘Sovereignty, Privacy and Contact Tracing Protocols’ in Linnet Taylor and others (eds), *Data Justice and COVID-19: Global Perspectives* (Meatspace Press 2020).

9 Yingzhi Yang and Julie Zhu, ‘Coronavirus Brings China’s Surveillance State out of the Shadows’ *Reuters* (Beijing/Hong Kong, 7 February 2020) <https://www.reuters.com/article/us-china-health-surveillance-idUSKBN2011HO> accessed 7 June 2020.

10 Emma Pinedo and Nathan Allen, ‘Deserted Streets, Talking Drones as Spain Imposes State of Emergency over Virus’ *Reuters* (Madrid, 16 March 2020) <https://www.reuters.com/article/us-health-coronavirus-spain-measures-idUSKBN213101> accessed 7 June 2020.

11 Paresh Dave, ‘Companies Bet on AI Cameras to Track Social Distancing, Limit Liability’ *Reuters* (Oakland, 27 April 2020) <https://www.reuters.com/article/us-health-coronavirus-surveillance-tech-idUSKCN22914R> accessed 7 June 2020.

12 Laure Wynants and others, ‘Prediction Models for Diagnosis and Prognosis of Covid-19: Systematic Review and Critical Appraisal’ [2020] BMJ <http://www.bmj.com/lookup/doi/10.1136/bmj.m1328> accessed 8 June 2020.

13 See Table 1—Data-driven tools against COVID-19.

17 Bobbie Johnson, ‘Nearly 40% of Icelanders Are Using a Covid App—and It Hasn’t Helped Much’ *MIT Technology Review* (11 May 2020) <https://www.technologyreview.com/2020/05/11/1001541/iceland-rakning-c19-covid-contact-tracing/> accessed 24 June 2020.

18 Julia Klaus, ‘Wo Die Corona-Warn-App Nicht Läuft’ (*ZDF*, 17 June 2020) <https://www.zdf.de/uri/2bb8ffd4-04a4-4705-a765-9d535d3a6866> accessed 8 July 2020.

19 ‘COVID-19 Digital Rights Tracker’ (*TOP10VPN*, 2020) <https://www.top10vpn.com/research/investigations/covid-19-digital-rights-tracker/> accessed 8 July 2020.

20 ‘Pandemic Big Brother’ <https://pandemicbigbrother.online> accessed 30 June 2020.

21 ‘Guidelines on the Use of Location Data and Contact Tracing Tools in the Context of the COVID-19 Outbreak’ (*European Data Protection Board*, 2020) 04/2020 <https://edpb.europa.eu/our-work-tools/our-documents/linee-guida/guidelines-042020-use-location-data-and-contact-tracing_en>.

22 Enrique Zapata and others, *The GovTech Index 2020: Unlocking the Potential of GovTech Ecosystems in Latin America, Spain and Portugal* (*CAF and Oxford Insights*, 2020) <scioteca.caf.com> accessed 7 July 2020.

23 ‘GDP (Current US$) by Country’ (*The World Bank*, 2019) <https://data.worldbank.org/indicator/NY.GDP.MKTP.CD> accessed 7 July 2020.

24 ‘Population, Total by Country’ (*The World Bank*, 2019) <https://data.worldbank.org/indicator/sp.pop.totl> accessed 7 July 2020.

25 ‘Coronavirus (COVID-19) Cases – Statistics and Research’ (*Our World in Data*) <https://ourworldindata.org/covid-cases> accessed 7 July 2020.

26 See ‘Human Rights of Persons with COVID-19’ (*Inter-American Commission on Human Rights*, 2020) Resolution 4/2020 <https://www.oas.org/en/iachr/decisions/pdf/Resolution-4-20-en.pdf> accessed 8 October 2020; ‘Joint Civil Society Statement: States Use of Digital Surveillance Technologies to Fight Pandemic Must Respect Human Rights’ (*Human Rights Watch*, 2 April 2020) <https://www.hrw.org/news/2020/04/02/joint-civil-society-statement-states-use-digital-surveillance-technologies-fight> accessed 6 July 2020; María Paz Canales, ‘Tecnología Contra La Pandemia: Derechos Fundamentales Mucho Más Que Daño Colateral’ (*Derechos Digitales*, 2 April 2020) <https://www.derechosdigitales.org/14355/tecnologia-contra-la-pandemia-derechos-fundamentales-mucho-mas-que-dano-colateral/> accessed 8 October 2020.

27 See ‘Ethical Considerations to Guide the Use of Digital Proximity Tracking Technologies for COVID-19 Contact Tracing’ (*World Health Organization – Global Health Ethics Unit*, 2020) <https://www.who.int/publications/i/item/WHO-2019-nCoV-Ethics_Contact_tracing_apps-2020.1>; ‘Guidelines on the Use of Location Data and Contact Tracing Tools in the Context of the COVID-19 Outbreak’ (n 21); Daniel Kahn Gillmor, ‘Principles for Technology-Assisted Contact-Tracing’ (*American Civil Liberties Union*, 2020) <https://www.aclu.org/report/aclu-white-paper-principles-technology-assisted-contact-tracing> accessed 25 June 2020; Andrew Crocker, Kurt Opsahl and Bennett Cyphers, ‘The Challenge of Proximity Apps For COVID-19 Contact Tracing’ (*Electronic Frontier Foundation*, 10 April 2020) <https://www.eff.org/deeplinks/2020/04/challenge-proximity-apps-covid-19-contact-tracing> accessed 8 July 2020.

28 ‘Coronavirus: Human Rights Need to Be Front and Centre in Response, Says Bachelet’ (*United Nations Human Rights – Office of the High Commissioner*, 6 March 2020) <https://www.ohchr.org/EN/NewsEvents/Pages/DisplayNews.aspx?NewsID=25668&LangID=E> accessed 6 July 2020.

29 ‘Joint Civil Society Statement: States Use of Digital Surveillance Technologies to Fight Pandemic Must Respect Human Rights’ (n 26); ‘Sociedad Civil Pide a Gobiernos de América Latina y El Caribe Que Tecnologías Digitales Aplicadas Ante La Pandemia COVID-19 Respeten Los DDHH’ (*TEDIC*, 20 March 2020) <https://www.tedic.org/sociedad-civil-pide-a-gobiernos-de-america-latina-y-el-caribe-que-tecnologias-digitales-aplicadas-ante-la-pandemia-covid-19-respeten-los-ddhh/> accessed 6 July 2020.

30 ‘COVID-19: States Should Not Abuse Emergency Measures to Suppress Human Rights’ (*United Nations Human Rights – Office of the High Commissioner*, 16 March 2020) <https://www.ohchr.org/EN/NewsEvents/Pages/DisplayNews.aspx?NewsID=25722&LangID=E> accessed 6 July 2020.

31 Andrej Zwitter and Oskar J Gstrein, ‘Big Data, Privacy and COVID-19 – Learning from Humanitarian Expertise in Data Protection’ (2020) 5 Journal of International Humanitarian Action 4–7.

32 Linnet Taylor and others (eds), *Data Justice and COVID-19: Global Perspectives* (Meatspace Press, London 2020).

33 Urs Gasser and others, ‘Digital Tools against COVID-19: Taxonomy, Ethical Challenges, and Navigation Aid’ [2020] The Lancet Digital Health 2 <https://linkinghub.elsevier.com/retrieve/pii/S2589750020301370> accessed 13 July 2020.

34 ‘Joint Civil Society Statement: States Use of Digital Surveillance Technologies to Fight Pandemic Must Respect Human Rights’ (n 26).

35 ‘Human Rights of Persons with COVID-19’ (n 26).

36 ‘Ethical Considerations to Guide the Use of Digital Proximity Tracking Technologies for COVID-19 Contact Tracing’ (n 27).

37 ‘Guidelines on the Use of Location Data and Contact Tracing Tools in the Context of the COVID-19 Outbreak’ (n 21).

38 Gillmor (n 27).

39 Crocker, Opsahl and Cyphers (n 27).

40 The requirement of voluntary use might negatively influence the uptake of the application by a population, which might compromise the effectiveness of the measure. However, the measures under analysis were developed in democratic countries, which suggests that individual autonomy and the rule of law are key principles that need to be respected, protected, and promoted by the executive.

41 Jessica Morley and others, ‘Ethical Guidelines for SARS-CoV-2 Digital Tracking and Tracing Systems’ [2020] SSRN Electronic Journal <https://www.ssrn.com/abstract=3582550> accessed 9 July 2020.

42 ‘Corona-Warn-App’ (*GitHub*, 2020) <https://github.com/corona-warn-app> accessed 12 July 2020; ‘DP-3T/Dp3t-App-Ios-Ch’ (*GitHub*, 2020) <https://github.com/DP-3T/dp3t-app-ios-ch> accessed 12 July 2020; ‘Corona Warn-App: Bericht Zur Datenschutz-Folgenabschätzung Für Die Corona-Warn-App Der Bundesrepublik Deutschland’ (2020) <https://www.coronawarn.app/assets/documents/cwa-datenschutz-folgenabschaetzung.pdf> accessed 13 July 2020.

43 ‘10 Requirements for the Evaluation of “Contact Tracing” Apps’ (*Chaos Computer Club (CCC)*, 6 April 2020) <https://www.ccc.de/en/updates/2020/contact-tracing-requirements> accessed 6 July 2020.

44 In the case of Norway, for instance, the Norwegian Data Protection Authority (DPA) decided to prohibit the operation of the government backed digital contact tracing app ‘Smittestopp’. The decision was made due to insufficient and non-transparent explanation of which data was used and collected by the app, which also included the use of location data from GPS. See ‘Bahrain, Kuwait and Norway Contact Tracing Apps a Danger for Privacy’ (*Amnesty International*, 16 June 2020) <https://www.amnesty.org/en/latest/news/2020/06/bahrain-kuwait-norway-contact-tracing-apps-danger-for-privacy/> accessed 12 July 2020; ‘The Norwegian Data Protection Authority Has Imposed a Temporary Ban on Smittestopp Contact Tracing Mobile Application’ (*Datatilsynet*, 2020) <https://www.datatilsynet.no/en/news/2020/the-norwegian-data-protection-authority-has-imposed-a-temporary-ban-on-smittestopp-contact-tracing-mobile-application/> accessed 9 December 2020.

45 Luisa Horwitz and others, ‘The Coronavirus in Latin America’ (*Americas Society/Council of the Americas*, 1 July 2020) <https://www.as-coa.org/articles/where-coronavirus-latin-america> accessed 3 July 2020.

46 Aislamiento Social Preventivo y Obligatorio 2020 [Decreto 297/2020].

47 Carolina Martínez Elebi, ‘Coronavirus: Datos, Salud Pública y Privacidad’ (Machine Intelligence Lab (MI Lab) del Centro de Estudios en Tecnología y Sociedad (CETyS) de la Universidad de San Andrés (UdeSA) 2020) <https://www.udesa.edu.ar/sites/default/files/milab.pdf>.

48 This also coincided with a name change to Cuidar. See ‘Sistema y Aplicación Cuidar’ (*Government of Argentina*) <https://www.argentina.gob.ar/jefatura/innovacion-publica/acciones-coronavirus/aplicacion-y-tableros-de-gestion> accessed 24 June 2020.

49 Ibid.

50 Ibid.

51 The Government established a list of activities that are exempted from isolation. For instance, such exceptions apply to Public Administration personnel working in essential sectors. The types of exempted activities may vary according to the province. See ‘Conocé si podés sacar el certificado y viajar en transporte público’ (29 June 2020) <https://www.argentina.gob.ar/circular/actividades-permitidas> accessed 9 December 2020; ‘Excepciones por provincia’ (23 April 2020) <https://www.argentina.gob.ar/coronavirus/aislamiento/provincias> accessed 9 December 2020.

52 To obtain the certificate is necessary to fill numerous personal information. The mandatory fields in the form require name, ID number, gender, telephone number, e-mail address, home address, and profession, among others. ‘Certificado de circulación – coronavirus COVID-19’ (*Argentina.gob.ar*, 27 May 2020) <https://www.argentina.gob.ar/circular> accessed 29 June 2020.

53 The ‘Terms and Conditions’ are not clear about the type of data that is being transmitted. See ‘Términos y Condiciones’ 3 <https://www.argentina.gob.ar/jefatura/innovacion-publica/acciones-coronavirus/aplicacion-y-tableros-de-gestion> accessed 8 December 2020.

54 According to the Article 19 of the Law No. 27.275, the *Agencia de Acceso a la Información Pública* act as the Data Protection Authority in Argentina. See Ley 27.275 – Derecho de Acceso a la Información Pública 2016.

55 ‘Guías para el tratamiento de datos personales’ (*Agencia de Acceso a la Información Pública*, 23 June 2020) <https://www.argentina.gob.ar/noticias/guias-para-el-tratamiento-de-datos-personales> accessed 8 October 2020.

56 Disposición 3/2020 2020.

57 The data collected through the app Cuidar is stored in the cloud service provided by Amazon Web Services, Inc. (AWS). ‘Informe 125’ 82 <https://www.argentina.gob.ar/jefatura/informes-al-congreso> accessed 2 July 2020.

58 ‘Los riesgos de la App Cuidar: algunas preocupaciones de derechos humanos’ 2–3 <https://amnistia.org.ar/los-riesgos-de-la-app-cuidar-algunas-preocupaciones-de-derechos-humanos/> accessed 8 October 2020.

59 Elebi (n 47).

60 Veale (n 8).

61 Ley 25.326 – Protección de Datos Personales 2000 12.

62 The app requires the following information: national ID number, e-mail address, and telephone number. Once this has been provided, it is possible to take the self-evaluation test.

63 ‘Los riesgos de la App Cuidar: algunas preocupaciones de derechos humanos’ (n 58).

64 Aplicación COVID-19 – Ministerio de Salud. Obligatoriedad de uso para toda persona que ingrese al país 2020 [Disposición 1771/2020].

65 ‘Cuidar Repository’ (*Github*, 25 May 2020) <https://github.com/argob/cuidar> accessed 30 June 2020.

66 Code source not available on 29 July 2020, when it was last consulted.

67 ‘Informe 125’ (n 57) 164.

68 Lei no 13.979, de 6 de Fevereiro de 2020. Dispõe sobre as medidas para enfrentamento da emergência de saúde pública de importância internacional decorrente do coronavírus responsável pelo surto de 2019. 2020 [13.979/2020].

69 The *Instituto Brasileiro de Geografia e Estatística* (IBGE) is the public agency responsible for conducting the census in the country, as well as the official collection of geographic, statistical, cartographic and environmental information.

70 Poder Executivo, Medida Provisória no. 954 2020 [MP 954/2020].

71 ‘IBGE Agradece a Adoção Da Medida Provisória No. 954’ (*Instituto Brasileiro de Geografia e Estatítica (IBGE)*, 17 April 2020) <https://www.ibge.gov.br/novo-portal-destaques.html?destaque=27475> accessed 30 June 2020.

72 ‘Comunicado Sobre Adoção Da Medida Provisória 954/2020’ (*Instituto Brasileiro de Geografia e Estatística (IBGE)*, 20 April 2020) <https://www.ibge.gov.br/novo-portal-destaques.html?destaque=27477> accessed 30 June 2020.

73 ‘Nota Da Coalizão Direitos Na Rede Sobre a Medida Provisória 954/20’ (*Direitos na Rede*, 20 April 2020) <https://direitosnarede.org.br/2020/04/20/nota-da-coalizao-direitos-na-rede-sobre-a-medida-provisoria-95420.html> accessed 30 June 2020.

74 ‘Ministra Suspende MP Que Prevê Compartilhamento de Dados Com o IBGE Por Empresas de Telecomunicações Durante Pandemia’ (*Supremo Tribunal Federal*, 24 April 2020) <http://www.stf.jus.br/portal/cms/verNoticiaDetalhe.asp?idConteudo=442090> accessed 6 July 2020.

75 Laura Schertel Mendes and Clara Inglesias Keller, ‘A New Milestone for Data Protection in Brazil’ [2020] Internet Policy Review <https://policyreview.info/articles/news/new-milestone-data-protection-brazil/1471>.

76 There was no clarity on the amount of people that would be actually interviewed by the *IBGE*, making the request to telecom operators to share data of millions of people with the organization unjustified.

77 Mendes and Keller (n 75); Luca Belli and Nicolo Zingales, ‘Brazilian Data Protection under Covid-19: Legal Certainty Is the Main Casualty’ (3 July 2020) <https://blogdroiteuropeen.com/2020/07/03/brazilian-data-protection-under-covid-19-legal-certainty-is-the-main-casualty-by-luca-belli-and-nicolo-zingales/> accessed 24 July 2020.

78 ‘Gobierno de Chile Crea App Para Prevenir Contagios de Coronavirus’ (*Ministerio de Vivienda y Urbanismo*) <https://www.minvu.cl/coronapp/> accessed 24 June 2020.

79 ‘FAQs CoronApp’ <https://coronapp.gob.cl/faqs.html> accessed 3 July 2020.

80 Ley 19.628 – Sobre Proteccion de la Vida Privada 1999.

81 Pablo Contreras and Michelle Bordachar, ‘Pandemia y datos sensibles’ (*CIPER*, 25 March 2020) <https://www.ciperchile.cl/2020/03/25/pandemia-y-datos-sensibles/> accessed 9 October 2020.

82 ‘Gobierno Lanza Aplicación ‘CoronApp’ Para Enfrentar El Coronavirus’ (*SENAMA – Ministerio de Desarrollo Social y Familia*, 20 April 2020) <http://www.senama.gob.cl/noticias/gobierno-lanza-aplicacion-coronapp-para-enfrentar-el-coronavirus> accessed 3 July 2020.

83 Ibid.

84 ‘Claveúnica’ <https://claveunica.gob.cl/> accessed 3 July 2020.

85 ‘Tecnología al Servicio de La Salud: ¡Gobierno Lanzó Aplicación Móvil ‘CoronApp’!’ (*División de Organizaciones Sociales*) <https://dos.gob.cl/tecnologia-al-servicio-de-la-salud-gobierno-lanzo-aplicacion-movil-coronapp/> accessed 3 July 2020.

86 ‘FAQs CoronApp’ (n 79).

87 ‘CoronApp: La Nueva Aplicación de Chile Para Combatir La Pandemia’ <https://digital.gob.cl/noticias/coronapp-la-nueva-aplicacion-de-chile-para-combatir-la-pandemia> accessed 3 July 2020.

88 Michelle Bordachar and Pablo Contreras, ‘Problemas de protección de los datos personales de la aplicación ‘CoronApp’’ (22 April 2020) <https://www.ciperchile.cl/2020/04/22/problemas-de-proteccion-de-los-datos-personales-de-la-aplicacion-coronapp/> accessed 9 October 2020.

89 ‘CoronApp: La Nueva Aplicación de Chile Para Combatir La Pandemia’ (n 87).

90 York Perry, ‘Coronavirus: Menos Del 1% de Chile Usa La CoronApp de Gobierno’ *FayerWayer* (28 May 2020) <https://www.fayerwayer.com/2020/05/coronavirus-chile-usa-coronapp/> accessed 24 June 2020.

91 See Table 2—Case studies evaluated according to guiding principles and best practices.

92 Morley and others (n 41).

96 ‘Exit through the App Store?’ (Ada Lovelace Institute 2020) <https://www.adalovelaceinstitute.org/wp-content/uploads/2020/04/Ada-Lovelace-Institute-Rapid-Evidence-Review-Exit-through-the-App-Store-April-2020-1.pdf> accessed 9 July 2020; Robert Hinch and others, ‘Effective Configurations of a Digital Contact Tracing App: A Report to NHSX’ (Pathogen Dynamics Group, Big Data Institute, Nuffield Department of Medicine, University of Oxford; Welcome Trust Centre for Ethics and the Humanities; IBM UK; UCL/Alan Turing Institute 2020) <https://github.com/BDI-pathogens/covid-19_instant_tracing/> accessed 9 July 2020.

97 ‘TraceTogether App’ <https://www.tracetogether.gov.sg/> accessed 11 July 2020.

98 Johnson (n 17).

99 ‘Ethical Considerations to Guide the Use of Digital Proximity Tracking Technologies for COVID-19 Contact Tracing’ (n 27); Marcello Ienca and Effy Vayena, ‘On the Responsible Use of Digital Data to Tackle the COVID-19 Pandemic’ (2020) 26 Nature Medicine 458.

100 Perry (n 90).

101 ‘CoronApp: La Inutilidad Del Atajo Tecnológico Desplegado Por El Gobierno y Sus Riesgos’ (n 95); Jessica Matus Arenas and others, ‘Declaración Sobre CoronaApp, Anunciada Por Gobierno de Chile Como Respuesta a COVID19’ (*Datos Protegidos*, 6 April 2020) <https://datosprotegidos.org/declaracion-de-fundacion-datos-protegidos-sobre-app-anunciada-por-gobierno-de-chile-como-respuesta-a-covid19/> accessed 11 July 2020.

102 ‘Unequal Disruptions: The Digital Divide During COVID-19 – AIIB Blog – AIIB’ <https://www.aiib.org/en/news-events/media-center/blog/2020/Unequal-Disruptions-The-Digital-Divide-During-COVID-19.html> accessed 24 July 2020; ‘Metade dos alunos fora da escola não tem computador em casa’ (*ONU News*, 21 April 2020) <https://news.un.org/pt/story/2020/04/1711192> accessed 24 July 2020.

103 Laura Robinson and others, ‘Digital Inequalities in Time of Pandemic: COVID-19 Exposure Risk Profiles and New Forms of Vulnerability’ (2020) 25 First Monday <https://firstmonday.org/ojs/index.php/fm/article/view/10845> accessed 12 July 2020.

104 Crocker, Opsahl and Cyphers (n 27).

105 ‘Digital Economy and Society Index Report 2020 – Human Capital’ (European Commission 2020) <https://ec.europa.eu/digital-single-market/en/human-capital> accessed 11 July 2020.

106 ‘The Inclusive Internet Index 2020’ (*EIU Inclusive Internet Index*, 2020) <https://theinclusiveinternet.eiu.com/explore/countries/performance?category=readiness> accessed 11 July 2020.

107 The concept of meaningful connection is concerned not only with internet access but also with the quality of the connection. In: ‘The Inclusive Internet Index 2020’ (*EIU Inclusive Internet Index*, 2020) <https://theinclusiveinternet.eiu.com/explore/countries/performance?category=readiness> accessed 11 July 2020.

108 The data resulting from the self-evaluation of symptoms through an app was analysed and used as evidence for a study that suggests that the loss of smell and taste can indicate COVID-19. Therefore, checking for these symptoms could become part of the screening routine. See Cristina Menni and others, ‘Real-Time Tracking of Self-Reported Symptoms to Predict Potential COVID-19’ (2020) 26 Nature Medicine 1037.

109 ‘Informe 125’ (n 57) 164.

110 Ibid.

111 ‘Certificado de circulación – coronavirus COVID-19’ (n 52).

112 ‘Transmission of COVID-19 by Asymptomatic Cases’ (*World Health Organization – Regional Office for the Eastern Mediterranean*, 6 July 2020) <http://www.emro.who.int/health-topics/corona-virus/transmission-of-covid-19-by-asymptomatic-cases.html> accessed 6 July 2020.

113 Don Weinland, ‘China’s Covid-19 QR Code Surveillance State’ (*Financial Times*, 7 May 2020) <https://www.ft.com/content/eee43c3e-8f7c-11ea-9b25-c36e3584cda8> accessed 22 July 2020.

114 Paul Mozur, Raymond Zhong and Aaron Krolik, ‘In Coronavirus Fight, China Gives Citizens a Color Code, With Red Flags’ (*The New York Times*, 1 March 2020) <https://www.nytimes.com/2020/03/01/business/china-coronavirus-surveillance.html> accessed 22 July 2020.

115 ‘‘Immunity Passports’ in the Context of COVID-19’ (*World Health Organization*, 24 April 2020) <https://www.who.int/news-room/commentaries/detail/immunity-passports-in-the-context-of-covid-19> accessed 8 October 2020.

116 ‘German Ethics Council Currently Advises against Covid-19 Immunity Certificates’ (Deutscher Ethikrat 2020) Press Release 07/2020 <https://www.ethikrat.org/en/press-releases/2020/german-ethics-council-currently-advises-against-covid-19-immunity-certificates/> accessed 8 October 2020.

117 United Nations, The Right to Privacy in the Digital Age 2016 [A/C.3/71/L.39/Rev.1].

118 Nicolo Zingales, ‘A Stronger Right to Data Protection During Pandemics? Leveraging the American Convention of Human Rights Against Governmental Inaction: A Brazilian Case-Study’ [2020] Revista de Direitos Fundamentais & Justiça (2020 Forthcoming).

119 Leonardo Parentoni and Henrique Cunha Souza Lima, ‘Protection of Personal Data in Brazil: Internal Antinomies and International Aspects’ [2019] SSRN Electronic Journal <https://www.ssrn.com/abstract=3362897> accessed 11 July 2020.

120 Dirceu Santa Rosa, ‘Brazilian Senate Postpones Its National Data Protection Law’ (*International Association of Privacy Professionals*, 6 April 2020) <https://iapp.org/news/a/brazilian-senate-postpones-its-national-data-protection-law/> accessed 11 July 2020; Belli and Zingales (n 77).

121 ‘Lei Geral de Proteção de Dados entra em vigor’ (*Senado Federal*) <https://www12.senado.leg.br/noticias/materias/2020/09/18/lei-geral-de-protecao-de-dados-entra-em-vigor> accessed 8 October 2020.

122 ‘CoronApp: La Inutilidad Del Atajo Tecnológico Desplegado Por El Gobierno y Sus Riesgos’ (n 95).

123 Ibid.

124 Crocker, Opsahl and Cyphers (n 27).

125 For its activation, the app requires numerous data, such as the RUN or passport number, telephone number, e-mail, name, age, geolocation, among others. In: ‘CoronApp: La Inutilidad Del Atajo Tecnológico Desplegado Por El Gobierno y Sus Riesgos’ (n 95).

126 After downloading the application, several data are required, such as the name, document number, gender, telephone number, among others. In: ‘Sistema y Aplicación Cuidar’ (n 48).

127 ‘Corona Warn-App: Bericht Zur Datenschutz-Folgenabschätzung Für Die Corona-Warn-App Der Bundesrepublik Deutschland’ (n 42).

## Notes

The authors thank Elizabeth Harvey and Alex Belloir for reviewing the manuscript.

